# Breast cancer mammographic diagnosis performance in a public health institution: a retrospective cohort study

**DOI:** 10.1007/s13244-017-0573-2

**Published:** 2017-10-04

**Authors:** Juliana M.R.B. Mello, Fernando P. Bittelbrunn, Marcio A. B. C. Rockenbach, Guilherme G. May, Leonardo M. Vedolin, Marilia S. Kruger, Matheus D. Soldatelli, Guilherme Zwetsch, Gabriel T. F. de Miranda, Saone I. P. Teixeira, Bruna S. Arruda

**Affiliations:** 1 Radiological Department, Hospital de Clínicas de Porto Alegre (HCPA), 2350, Ramiro Barcelos St. Second floor, Porto Alegre, 90035-903 Brazil; 2 Neuroradiology Department, Hospital Moinhos de Vento, Porto Alegre, Brazil

**Keywords:** Mammography auditing, Breast cancer, Quality assurance, SISMAMA, Public health

## Abstract

**Objectives:**

To evaluate the quality assurance of mammography results at a reference institution for the diagnosis and treatment of breast cancer in southern Brazil, based on the BIRADS (Breast Imaging Reporting and Data System) 5th edition recommendations for auditing purposes.

**Materials and methods:**

Retrospective cohort and cross-sectional study with 4502 patients (9668 mammographies)) who underwent at least one or both breast mammographies throughout 2013 at a regional public hospital, linked to a federal public university. The results were followed until 31 December 2014, including true positives (TPs), true negatives (TNs), false positives (FPs), false negatives (FNs), positive predictive values (PPVs), negative predictive value (NPV), sensitivity and specificity, with a confidence interval of 95%.

**Results:**

The study showed high quality assurance, particularly regarding sensitivity (90.22%) and specificity (92.31%). The overall positive predictive value (PPV) was 65.35%, and the negative predictive value (NPV) was 98.32%. The abnormal interpretation rate (recall rate) was 12.26%.

**Conclusions:**

The results are appropriate when compared to the values proposed by the BIRADS 5th edition. Additionally, the study provided self-reflection considering our radiological practice, which is essential for improvements and collaboration regarding breast cancer detection. It may stimulate better radiological practice performance and continuing education, despite possible infrastructure and facility limitations.

***Main Messages*:**

*• Accurate quality performance rates are possible despite financial and governmental limitations.*

*• Low-income institutions should develop standardised teamwork to improve radiological practice.*

*• Regular mammography audits may help to increase the quality of public health systems.*

## Introduction

The main goal of breast cancer screening is to reduce mortality rates through early detection and proper treatment [1–6]. Great effort has been made in the last years by the international scientific community, particularly in the radiology field, to achieve this goal [7–9]. Recently, a systematic review showed a decrease of at least 20% for the mortality rate due to the use of mammography for breast cancer screening [10]. Another study attributes most of the decrease in the breast cancer mortality rate of at least 38% since 1990 to early mammography detection [11]. Nevertheless, uncertainties remain about the magnitude of overdiagnosis associated with different possible screening strategies [10]. Meanwhile, the main imaging modality for breast cancer screening is still mammography [12, 13].

As a method of standardising the process of reporting mammograms and also with the objective of facilitating data collection, the American College of Radiology (ACR) created the lexicon published in the atlas “Breast Imaging Reporting and Data System (BIRADS)”, which is used worldwide as a single and united system for the breast imaging radiology subspecialty [13]. It provides a guide to mammography audits and performance measures [14].

Mammography auditing assumes a relevant role when discussing the quality of breast imaging, whether regarding the radiologist’s interpretation or the quality of the images taken [1, 15, 6]. The majority of developed countries, such as the USA, have a federal standardisation for evaluating the personal performance of a radiologist as well as the imaging centres [9, 16, 17]. In Europe, for example, there is the Dutch Reference Centre of Screening, which conducts triennial audits of 17 mammography performing centres [18]. In this European programme, they showed increased detection rates and increased sensitivity (up to 71.6%) over the last audit series performed from 1996 to 2013 [18].

In Latin America, some underdeveloped countries are trying to implement policies to address the growing incidence of breast cancer, especially in Brazil and Mexico, and also to promote an early breast cancer detection strategy [19]. In Asia, in Taiwan, there is a study showing improvement in their performances, with a sensitivity rate varying from 79.6% to 87% and a specificity rate varying from 90.5% to 91.1% [20]. Meanwhile, even in developed countries, like the USA, many radiologists report their accuracy goals are below the published desirable benchmarks [21]. This information enhances the need for specific attention to the mammography audit performance and breast imaging education worldwide.

However, in Brazil, most imaging centres do not perform an internal audit to compare their rates with the BIRADS recommendations. There is a Brazilian government task force to solve this problem, and it has created a national standardised programme, called SISMAMA (since 2009). This programme will help institutions and hospitals all over the country collect more accurate data and facilitate performance rate evaluations [22].

To encourage good radiological practice in our service either individually or as a group of radiologists and to determine whether our practice is in accordance with the international performance standards, we performed a retrospective cohort observational and cross-sectional study for the diagnostic mammography results in our hospital from 2013.

## Materials and methods

We present a retrospective cohort observational and cross-sectional study, with all the BIRADS mammography results from 1 January 2013 until 31 December, 2013, in a regional reference public hospital, which were submitted to an internal audit. The BIRADS mammography results were analysed using a national software, provided by the Brazilian National Cancer Institute (INCA), called SISMAMA, which aims to nationalise all mammography results as a way to congregate more data.

A total of 9668 mammographies were performed in 4502 patients between the ages 28 and 92 years in one or both breasts in 2013 in our hospital. The mammography results, under approval from our regional and hospital ethics committee, were submitted to an internal audit. We considered all the mammographies as “diagnostic”, because we always have at least one radiologist to evaluate the images before releasing the patient in our service, and also because as we are a reference centre for breast imaging in public health, almost all our patients have been previously referred by many smaller regional health centres as symptomatic patients.

All the mammographies that had positive results (classified as BIRADS 0, 4 and 5) were followed to verify further examinations, surgical procedures and biopsies performed for 1 year after the mammogram had been taken, until 31 December 2014. Mammographies classified as BIRADS 1, 2 and 3 were considered negative and all results of BIRADS 6 were also not considered for audit purposes, because the BIRADS 5th edition recommends doing so in diagnostic mammography audits.

After selecting all considered BIRADS-positive results (BIRADS 0, 4 and 5), which added up to 664 mammographies, we reviewed the mammographic reports, breast ultrasound reports, anatomopathologic biopsy results (guided by either ultrasound or stereotactic imaging-guided procedures to investigate suspicious cluster microcalcifications) and anatomopathologic results from surgery procedures.

The formulas and calculus used were from the last 5th editionof the BIRADS atlas for auditing purposes. The performance measures were calculated based on the numbers of true-positive (TP), false-positive (FP), true-negative (TN), false-negative (FN) and total mammographic examinations (N) summed over the year of 2013.

The performance measures were calculated as follows:

- Sensitivity = TP/(TP + FN);
- Specificity = TN/(TN + FP);
- Positive predictive value (PPV) = TP/(TP + FP);
- Negative predictive value (NPV) = TN/(TN + FN);
- Positive predictive value 2 (PPV2 or biopsy recommended) = TP/(number of diagnostic examinations recommended for tissue diagnosis);
- Positive predictive value 3 (PPV 3 or biopsy performed) = TP/(number of biopsies performed).

True positive (TP) is defined as a tissue diagnosis of cancer within 1 year after a positive examination. True negative (TN) is defined as no known tissue diagnosis of cancer within 1 year after a negative examination. False negative (FN) is defined as tissue diagnosis of cancer within 1 year after a negative examination. False positive (FP) is defined as no known tissue diagnosis of cancer within 1 year after a positive examination.

Our standard comparisons were the values proposed by the BIRADS 5th edition recommendations for diagnostic auditing purposes (table presented on page 593) and also by the Breast Cancer Surveillance Consortium (BCSC) Benchmarks results available in the last BIRADS 5th edition (table presented on page 591) to check if the radiological practice was accurate for a breast imaging reference centre. The BCSC Benchmarks results we used to compare with our data were collected from a table presented in the 5th BIRADS Atlas Edition (page 591), comprising 401,572 diagnostic mammography examinations taken between 1996 and 2005, collected from 153 mammography facilities and 741 interpreting physicians that demonstrate a geographically and ethnically representative sample of the US population [13].

Our mammographic results included reports from four different radiologists (with 5 to 20 years of experience in breast imaging), which were retrospectively accessed.

This was a single-centre study with two digital mammography machines (Siemens Mammomat Inspiration), which were recently acquired by the hospital, and five ultrasound machines (Philips and Aloka). These machines were used to perform all mammographies and any complementary examinations that needed to be carried out, such as ultrasounds, ultrasound-guided biopsies or further investigations that were needed because of the mammography results.

No patient had any known direct or prompt adverse event due to this study, besides undergoing a mammogram and being exposed to the examination radiation. This radiation mean (entrance surface dose) was 2.6 mGy during 2013 in our service, depending on the thickness and composition of the breast.

STATA software (StataCorp 2013; Stata: Release 13 Statistical Software) was chosen for statistical analysis. We calculated the overall positive predictive value (PPV), negative predictive value (NPV), sensitivity and specificity of our mammography results with 95% of confidence intervals (CI).

## Results

The BIRADS distribution of the 9668 mammography examinations (accounting a mammography for each breast separately) during 2013 in our institution is shown in Table 1.

**Table 1:** Number of mammographies and their distribution percentages in the BIRADS categories

Mammography distribution into the BIRADS categories in our institution		
BIRADS categories	No. of mammographies	% of mammographies
**Category 0**	478	4.94%
**Category 1**	1641	16.97%
**Category 2**	6927	71.65%
**Category 3**	235	2.43%
**Category 4**	137	1.42%
**Category 5**	49	0.51%
**Category 6**	201	2.08%
**Total**	9668	100.00%

The distribution of the anatomy composition of the breast, following the BIRADS recommendations, is shown in Table 2. The anatomy of the breast (divided into extremely dense, heterogeneously dense, scattered fibroglandular tissue and entirely fat) was divided into each BIRADS category, 0, 1, 2, 3, 4, 5 and 6, during 2013 at our institution.

**Table 2:** Breast anatomy composition distribution into the BIRADS categories during 2013 at our institution

Breast anatomy composition into the BIRADS categories at our institution			
BIRADS category	Breast anatomy composition	Total	%
**0**	Entirely fat	13	2.72%
	Extremely dense	134	28.03%
	Scattered areas of figroglandular tissue	155	32.43%
	Heterogeneously dense	176	36.82%
**BIRADS 0 total**		**478**	
**1**	Entirely fat	118	7.19%
	Extremely dense	374	22.79%
	Scattered areas of figroglandular tissue	598	36.44%
	Heterogeneously dense	551	33.58%
**BIRADS 1 total**		**1641**	
**2**	Entirely fat	571	8.27%
	Extremely dense	1342	19.43%
	Scattered areas of figroglandular tissue	2783	40.29%
	Heterogeneously dense	2211	32.01%
**BIRADS 2 total**		**6907**	
**3**	Entirely fat	11	4.68%
	Extremely dense	72	30.64%
	Scattered areas of figroglandular tissue	76	32.34%
	Heterogeneously dense	76	32.34%
**BIRADS 3 total**		**235**	
**4**	Entirely fat	5	3.65%
	Extremely dense	46	33.58%
	Scattered areas of figroglandular tissue	33	24.09%
	Heterogeneously dense	53	38.69%
**BIRADS 4 total**		**137**	
**5**	Entirely fat	3	6.12%
	Extremely dense	12	24.49%
	Scattered areas of figroglandular tissue	24	48.98%
	Heterogeneously dense	10	20.41%
**BIRADS 5 total**		**49**	
**6**	Entirely fat	9	4.48%
	Extremely dense	62	30.85%
	Scattered areas of figroglandular tissue	65	32.34%
	Heterogeneously dense	65	32.34%
**BIRADS 6 total**		**201**	

We selected the categories 0, 4 and 5, which totaled 664 mammographies in 2013, as recommended by the BIRADS 5th edition, to perform an internal audit of diagnostic mammograms.

As the BIRADS 5th edition says, category 0 means the radiologist who did the report believes the mammogram finding will need an additional evaluation, such as an additional examination or breast ultrasound. Categories 4 and 5 mean the radiologist who did the report believes the mammogram finding will need further histopathological evaluation, such as a core biopsy. The radiologist decision between categories 4 or 5 depends on his/her analysis of the mammogram finding and how likely it is to be a breast cancer or not. How the radiologist makes his/her decision depends on training, education, experience and the BIRADS 5th edition recommendations [13].

There were 83 true positives (TP), 44 false positives (FP), 9 false negatives (FN) and 528 true negatives (TN) when considering the sample of 664 mammographies in the categories 0, 4 and 5.

**Fig. 1:**
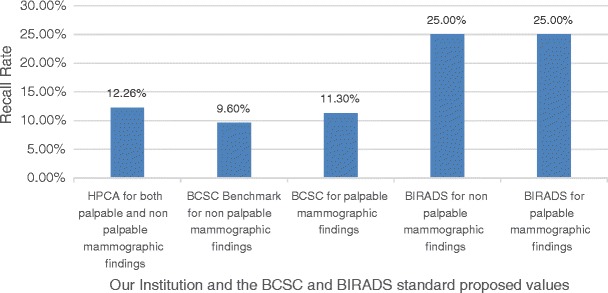
Percentages of abnormal interpretation/recall rate

Our cancer detection rate was 19.34% and the abnormal interpretation rate (or recall rate) was 12.26%, as shown in Fig. 1. The BCSC Benchmarks (for either palpable or non-palpable mammographic findings) show similar values to our institution, although smaller. Nevertheless, our institution recall rate value (for both palpable and non-palpable findings) is smaller than the recommended BIRADS (for either palpable and non-palpable findings) standards.

**Fig. 2:**
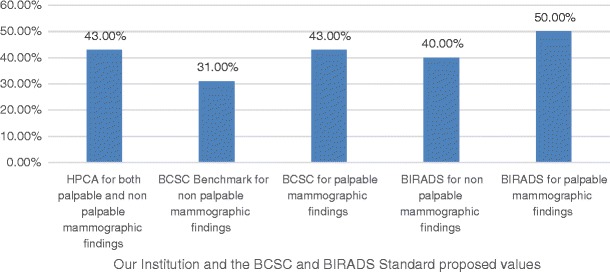
PPV2 showing how frequently the performed biopsies indicate cancer

Our positive predictive value 2 (recommendation for tissue diagnosis or PPV2) was 43%, as illustrated in Fig. 2. This is an important subanalysis because it shows the accuracy radiologists expect for recommending a histopathological correlation for a suspicious mammographic finding.

**Fig. 3:**
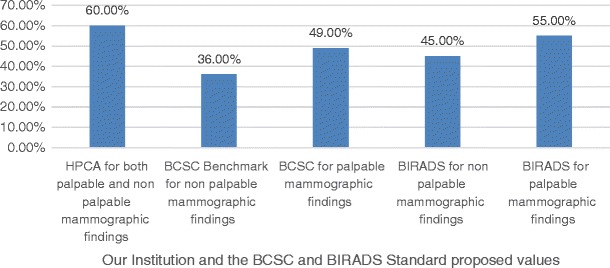
PPV3 showing how frequently performed biopsies indicated cancers

Positive predictive value 3 (biopsy performed or PPV3) was 60%, as shown in Fig. 3.

Usually the PPV3 is higher than the PPV2, since the biopsies that will actually be done will probably show positive results for cancer. Regarding this topic, our results showed the same outcome. As BIRADS recommends, PPV3 is a more accurate indicator of cancer status, and PPV2 is more accurate for interpretive performance [13].

**Fig. 4:**
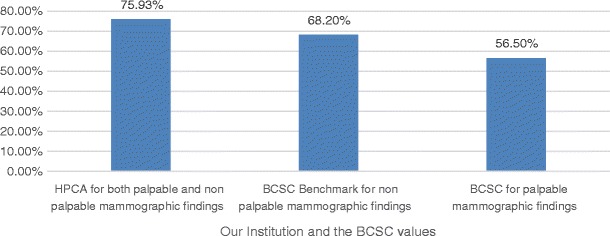
Percentages of invasive cancers with negative axillary lymph nodes

Our rate of negative axillary lymph nodes was 75.93%, as shown in Fig. 4.

**Fig. 5:**
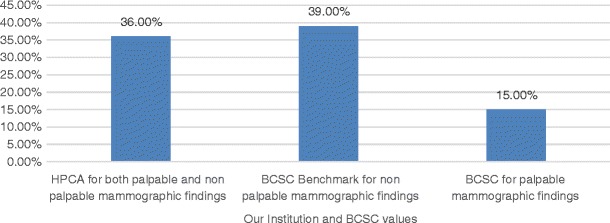
Percentages of minimum invasive cancers (<1.0 cm) or DCIS

The minimum cancer rate (invasive cancer with 1.0 cm or less or DCIS, ductal carcinoma in situ) was 36%, as illustrated in Fig. 5.

The sensibility of our diagnostic mammograms in 2013 was 90.22% (95% CI 82.24%–95.43%) and the specificity was 92.31% (95% CI 89.91% to 94.36%).

The positive predictive value (PPV) was estimated in 65.35% (95% CI 58.49%–71.63%).

The negative predictive value (NPV) was estimated in 98.32% (95% CI 96.93%–99.09%).

## Discussion

As a limitation of this study, the comparison between our rates and the BCSC rates regarding “minimally invasive cancers or DCIS” and “invasive cancers with negative axillary lymph nodes” could not be done using proper additional statistical analysis. The main reasons were limited access to BCSC data and also because their sample size for the table presented on page 591 of the last 5th edition of BIRADS (401,572 diagnostic mammograms between 1996 to 2005) had a distinct difference from our sample size (9668 mammograms during 2013). This distinct difference between the sample sizes compared could have led us to false results. However, if we consider a confidence interval of 95%, our sample size should be accurate to compare our proportions and BCSC proportions, but we could not calculate a specific *p*-value for these comparisons.

We had a total of 58 losses, whose data records were not available in the hospital electronic system, mainly because these 58 patients (8.7% of our sample of 664 positive mammography results) had private health care systems, which did not allow them to be classified into the hospital's public health care electronic system.

Our cancer detection rate was 19.34%, close to the minimum 20% required for diagnostic mammograms according to BIRADS. This result is possibly related to a minor skewed distribution of cases mainly due to the few screening mammograms in the total number of 9668 mammographies selected and considered as diagnostic in our study. Additionally, it may be secondary to the fact that our SISMAMA nationalised software does not have a digital option to separate systematic screening from diagnostic mammograms. This may result in some minor skewed distribution of the data when considering the cancer detection rate, which would certainly not invalidate our results.

The abnormal interpretation rate (or recall rate) was 12.26%, below the BCSC Benchmarks for diagnostic mammograms (13.3%) and adequate for the value recommended by BIRADS (8–25% for non-palpable findings or 10–25% for palpable findings), as shown in Fig. 1. There are many reasons for this lower recall rate, such as the prompt review of the images by a breast radiologist immediately after they are taken, allowing performing additional mammography examinations when the patient is still inside the service. Other reasons are the experience and training of the radiologist's team.

Our positive predictive value 2 (recommendation for tissue diagnosis or PPV2) was 43%, which is exactly the same value as in BCSC for palpable findings (43%), above the value of BCSC Benchmarks for non-palpable findings (31%), and near the value requested by BIRADS for non-palpable findings (15–40%) and by BIRADS for palpable findings (25–50%). This PPV2 shows that our recommendations for biopsies are accurate.

Our positive predictive value 3 (biopsy performed or PPV3) was 60%, which was above all the standard comparisons: BCSC Benchmarks for non-palpable findings (36%), BCSC for palpable findings (49%), BIRADS for non-palpable findings (20–45%) and BIRADS for palpable findings (30–55%). We believe the reason for this high PPV3 is the fact that most of symptomatic patients were referred by previous smaller health care centres to our hospital, which centralise most of the patients who will need to be treated for breast cancer using the national public health system. Moreover, we have patients who already had undergone some previous screening before having a mammography in our hospital. Another reason to consider is that many patients are already symptomatic at the time of the breast cancer diagnosis in south Brazil, as well as in other low-income regions worldwide. This situation reflects the fact that some patients do not have ideal social and economic possibilities for having a mammography performed in an earlier stage of the disease.

Our rate of negative axillary lymph nodes was 75.93%, above the BCSC Benchmarks for non-palpable findings (68.2%) and BCSC in palpable findings (56.5%). This is another positive result, which may contribute to lower morbidity and mortality rates for the patients’ outcomes.

The minimum cancer rate (invasive cancer with 1.0 cm or less or DCIS, ductal carcinoma in situ) was 36%, near the BCSC Benchmarks for non-palpable findings (39%) and above the BCSC for palpable findings (15%). For the minimum cancer rate, BIRADS does not determine a fixed number, because it is implicit that the higher it is, the better it will be for both patients and professionals related to the treatment of these patients.

The sensitivity of our diagnostic mammograms in 2013 was 90.22%, above all the standard comparisons: the BIRADS recommendation for non-palpable findings (80% or higher), BIRADS for palpable findings (85% or higher), BCSC Benchmarks for non-palpable findings (83.1%) and BCSC in palpable findings (87.8%).

The specificity of our diagnostic mammograms in 2013 was 92.31%, adequate for all the standard comparisons: BIRADS recommendations for non-palpable findings (80–95%), BIRADS recommendations for palpable findings (83–95%), BCSC Benchmarks for non-palpable findings (92.2%) and BCSC for palpable findings (93.2%).

As shown in the results, most of our performance rates regarding mammography reports are similar to (sometimes better than) the values proposed by BIRADS as good radiological practice. The sensitivity and specificity of our diagnostic mammographies are adequate for all the standard comparisons, moving the values of our institution to the international aim of reducing the mortality and morbidity related to breast cancer.

Nevertheless, this study has a higher internal than external validity mainly because it is a single-centre study. Despite this, it can stimulate good radiological practice in public health institutions worldwide, even with poorer general infrastructure conditions when compared to the available facilities in reference institutions in developed countries.

Our secondary goal in doing the study was to encourage radiologists to be curious and try to look for their overall performance and self-evaluation (either individually or as a team). As discussed in the literature, the feedback to the team of radiologists as well as the moments of self-reflection and insights in recall behaviour on mammograms is extremely valuable. By doing this, other institutions also may improve their internal collaboration process towards meeting the patients’ needs. Additionally, we had more understanding about how we can achieve higher scores and solve some problems concerning data collection.

Knowing the teamwork performance is essential to discovering different means of improvement and to improving the radiological medical practice. By pointing out the limitations and also our good results, the current study had a positive impact on our daily practice, especially regarding our focus on enhancing the breast cancer diagnosis detection when performing mammograms. In the future, we intend to implement arrangements for collecting data, analysing results and also concentrating more efforts on continuing medical education for all radiologists.

## Conclusion

This study is an internal audit in a regional reference institution for breast cancer diagnosis, treatment and follow-up in an underdeveloped country. We performed a retrospective observational and cross-sectional cohort study to analyse our radiological practice accuracy considering mammography reports. Despite possible limitations, our results showed high-quality rates, demonstrating that standardised teamwork can promote the quality of public health systems worldwide. Additionally, it may encourage radiologists to seek performance feedback and continuing medical education, thus resulting in better outcomes for the patients.

## Abbreviations

ACR: American College of Radiology
BIRADS: Breast Imaging Reporting and Data System
BCSC: Breast Cancer Surveillance Consortium
CI: Confidence interval
FN: False negative
FP: False positive
HCPA: Hospital de Clínicas de Porto Alegre (Porto Alegre’s Clinical Hospital)
INCA: Instituto Nacional do Câncer (Cancer National Institute)
PPV: Positive predictive value
NPV: Negative predictive value
SISMAMA: Sistema de Informação do Câncer de Mama (Breast Cancer Information System)
TN: True negative
TP: True positive
